# A Multi-Domain Intervention Protocol for the Potential Reversal of Cognitive Frailty: “WE-RISE” Randomized Controlled Trial

**DOI:** 10.3389/fpubh.2020.00471

**Published:** 2020-09-03

**Authors:** Resshaya Roobini Murukesu, Devinder Kaur Ajit Singh, Suzana Shahar, Ponnusamy Subramaniam

**Affiliations:** ^1^Physiotherapy Programme and Centre for Healthy Aging and Wellness, Faculty of Health Sciences, Universiti Kebangsaan Malaysia, Kuala Lumpur, Malaysia; ^2^Dietetic Program and Centre for Healthy Aging and Wellness, Faculty of Health Sciences, Universiti Kebangsaan Malaysia, Kuala Lumpur, Malaysia; ^3^Health Psychology Programme and Centre for Healthy Aging and Wellness, Faculty of Health Sciences, Universiti Kebangsaan Malaysia, Kuala Lumpur, Malaysia

**Keywords:** cognitive frailty, frailty, cognitive impairment, multi-domain intervention, community dwelling, older adults

## Abstract

Following the rapid increase of the aging population, health promotion and prevention of physical disability and dementia in older persons are essential for healthy aging. For example, there may be a potential to prevent or reverse cognitive frailty, the co-existence of both physical frailty and cognitive impairment in older persons. However, evidence-based interventions targeting the prevention or potential reversibility of cognitive frailty among community dwelling older adults are scarce. In this paper, we described the rationale, development and delivery of a multi-domain intervention comprising multi-component physical exercise prescription, cognitive training, dietary counseling and promotion of psychosocial support, called the WE-RISE trial. The aim of WE-RISE intervention is to potentially reverse cognitive frailty. This is a two-armed, single blinded, randomized controlled trial conducted over a duration of 6 months, at senior citizen activity centers within the Klang Valley, Malaysia. Ambulating, community dwelling older adults aged 60 years and above with cognitive frailty are randomized into two groups; (1) intervention group: which receives an instructor based “WE-RISE” intervention for the first 3 months, and then a home-based “WE-RISE at Home” intervention for the following 3 months; (2) control group: usual care with no modifications to their daily routine. Primary outcome is cognitive frailty status and secondary outcome include physical function, cognitive performance, nutritional status, psychosocial status and quality of life which are obtained during baseline screening and subsequent follow ups at 3rd and 6th month. Description of the intervention is done using the template for intervention description and replication (TIDieR) checklist. This trial protocol has received approval from Research Ethics Committee of Universiti Kebangsaan Malaysia (UKM PPI/111/8/JEP-2018-558) and the Department of Social Welfare Malaysia (MyResearch Reference: JKMM 100/12/5/2: 2018/405). Trial registration number: ACTRN12619001055190.

## Introduction

The average lifespan of an individual has globally increased. Malaysia is expected to be labeled as an “aged nation” by the year 2035 with 15% of its' country comprising of older persons (1) Older persons remain predisposed to adverse health outcomes resulting in reduced quality of life and increased cost of healthcare (2). Frailty and cognitive impairment have been enlisted as two of the four modern “giants of geriatrics,” followed by sarcopenia and anorexia of aging (3).

Physical frailty, a prevalent clinical syndrome is manifested due to age-related degeneration of multiple systems leading to rapid health decline in older persons (4, 5). Older persons with frailty are more vulnerable and are susceptible to events such as falls, physical disability, disruption of functional independence, prolonged hospitalization, institutionalization, and fatality (5, 6). Recognized as an intermediate stage between robust and disability among older persons, frailty can be identified with the presence of unintentional weight loss, fatigue, weakness, slow motor performance, and physical inactivity as outlined in the Cardiovascular Health Study (CHS) (6).

While, mild cognitive impairment (MCI) is a symptomatic, pre-dementia phase, characterized by self-reported clinical concern, and objective memory deficits, but without functional decline (7). The occurrence of cognitive decline has been associated with vascular disease, metabolic disorder, trauma, infectious diseases, depression and polypharmacy (8). Marked changes in cognitive function may commence as early as between 3 and 7 years preceding the diagnosis of MCI (9). Hence MCI, a prodromal symptom of dementia is a primary target for early intervention to prevent or delay the progression into an irreversible state of cognitive impairment (10).

Amongst the Malaysian community dwelling older population, the prevalence of frailty and pre-frailty was reported to be 8.9 and 61.7%, respectively (11), whereas MCI is prevalent at 16% (12). Prevalence of frailty and pre-frailty has been reported to be much higher amongst institutionalized older persons at 40.7 and 56.6%, respectively, with cognitive impairment as a predictor (13). The stark contrast in prevalence between community dwelling and the more vulnerable institutionalized population is attributed to poorer health outcomes; mainly severe physical and cognitive impairments (14). There is a cyclic relationship between physical frailty and cognitive impairment, whereby the disintegration of one construct is likely to result in the consequent declination of the other (15, 16). Aimed at coalescing both constructs, the “cognitive frailty” syndrome among older adults was established in 2013 (17). Cognitive frailty is defined as a “heterogeneous clinical manifestation characterized by the simultaneous presence of physical frailty and diagnosis of cognitive impairment excluding the presence of concurrent Alzheimer's disease (AD) or other dementias” (17).

Prevalence of cognitive pre-frailty and cognitive frailty among community dwelling Malaysian older population was reported at 37.4 and 2.2%, respectively (18). Results from a longitudinal study reported the incidence rate of cognitive frailty in Malaysia at 7.1 per 100 person-years among older adults who were non-cognitively frail at baseline (19). The rate of incidence increased with increasing age among older adults aged 75 years and above, whereby it doubles every 10 years; estimated at 13.34 per 100 person-years (19). Research concerning cognitive frailty in the Malaysian context is a recent development and there is currently no available local intervention addressing this prevalent condition.

At present, physical frailty and cognitive impairment are often separately studied and addressed, despite the evidence that both are correlated (17). Although, cognitive frailty has been deduced to be “potentially reversible,” a specific intervention targeted at addressing this condition in the community and home based setting remains unestablished (20). Thus, we aim to examine the effectiveness of a newly developed multi-domain intervention for possible reversal of cognitive frailty among Malaysian community dwelling older persons.

## Methods and Materials

### Rationale for the Development of a Multi-Domain Intervention

The rationale for the development of this intervention is to address the multi-factorial predictors of cognitive frailty. This include poor physical fitness, functional dependence, depression, lack of social support, and nutritional deficiency (18, 19). There is substantial evidence advocating for the development of an intervention employing a multi-domain approach in the attempt to delay or reverse this condition; rather than a singular approach (21).

Older persons living with lower socioeconomic status are at higher risk of being frail and care dependent due to malnutrition, poor physical health practices, inevitably subjecting them to disability, and mortality (22). Moreover, these older adults are more inclined to have decreased cognitive function possibly due to lower education levels (22). Within the community-based rehabilitation settings, there is yet to be a complimentary, self-sustaining, low cost intervention for these older persons. Hence, the development of this multi-domain intervention aims to bridge this existing gap.

### Components of the Multi-Domain Intervention

Evidence of the effectiveness of a multi-domain intervention in addressing cognitive frailty is currently unavailable. However, multi-domain intervention approach has been utilized with positive outcomes for physical frailty and cognitive impairment separately. For example, in the study by Ng et al. (23), a multidomain approach combining nutritional, physical and cognitive interventions significantly reduced frailty among pre-frail and frail older adults. Similar intervention has also been aimed at delaying cognitive impairment among older persons at risk of cognitive decline in the 2-year Finnish Geriatric Intervention Study to Prevent Cognitive Impairment and Disability (FINGER) trial which consisted of nutritional guidance, exercise, cognitive training and social stimulation as well as monitoring metabolic and vascular risk factors (24). It is noteworthy that these interventions were client-tailored and not community-based. Inclusion of physical activity, cognitive training, nutritional and dietary guidance, emotional recovery, and social support via a multi-faceted approach are the recommendations for potential reversibility of the cognitive frailty status (20, 25). These evidence and recommendations served as the foundation for the development of our current multi-domain intervention for reversible of cognitive frailty.

Exercise training has been demonstrated to be beneficial in improving frailty symptoms and cognitive function, in addition to being cost effective (26, 27). The evidence corroborates with the existing notion that exercise has the potential to reverse frailty by improving physical function (28). Similarly, exercise, specifically aerobic training was concluded to be effective in sustaining cognitive function and may delay the occurrence of cognitive decline in a systematic review (27).

While, cognitive training is beneficial in training or re-training relatively well-defined cognitive abilities such as information processing, attention, memory or problem solving via the concept of neuroplasticity (29). Taking this concept into consideration, cognitive based exercises involving guided practice structured tasks have been incorporated into interventions to enhance cognitive function (29, 30). Although, lacking high impact evidence, cognitive training has been used in combination with other domains of intervention (physical activity, socialization or healthy diet) to reduce the risk of cognitive decline and progression into dementia (29, 31).

In regard to nutritional interventions, it is important in addressing cognitive frailty as malnutrition is a contributor to the onset and progressive worsening of cognitive frailty and other related co-morbidities (25). Deficiency of protein, vitamin D, vitamin B12, inadequate calorie intake or even over-eating in middle age have been enlisted as several factors leading to sarcopenia, weight loss, fatigue and cognitive decline among older adults (18, 25, 32). There is evidence to suggest that nutritional interventions promoting a continuous practice of balanced dietary patterns, could delay cognitive frailty (25). Additionally, combination intervention such as exercise and nutrition is strongly recommended as compared to nutritional intervention alone among frail and cognitively impaired older adults (33).

Generally, healthy or successful aging include physical, mental, and psychosocial well-being (34). Factors such as positive self-esteem, self-achievement, self-worth, and self-efficacy are associated with positive health outcomes among older persons (34). An association between poor psychosocial status, cognitive impairment, and physical frailty has been established (21). Notably, incorporating elements of fun, social inclusion, and enjoyment in older persons with frailty resulted in improved frailty scores, delaying functional decline, improved quality of life as well as psychosocial well-being (35). Promotion of social interaction via group-based interventions have also shown favorable outcomes amongst older persons with cognitive impairment and frailty (21, 36).

### Development and Selection of Specific Intervention

An evidence-based exercise program for older adults with cognitive frailty is not available. Thus, we developed a multi-component exercise program based on the combination of the available evidence. Firstly, the components of aerobic exercise, progressive resistance training, balance and flexibility were selected as it addresses the frailty criterion of weakness, slow motor performance, low physical activity, and fatigability (26, 37). Next, we benchmarked the evidence in an umbrella systematic review of systematic reviews (total of 58 RCTs) evaluating the effectiveness of exercise-based interventions among those with pre-frailty and frailty (26). In this review, it was concluded that multi-component exercise training was the most effective form of exercise-based intervention among pre-frail and frail older persons as it improved physical performance, specifically muscle strength, gait speed, and balance (26). The proposed outline of a multi-component exercise program targeting older persons with frailty should encompass progressive resistance training, aerobic, balance, and flexibility training. The frequency of exercise sessions was suggested up to thrice weekly for a duration of between 45 and 60 min per session at moderate to high intensity. Effective exercise program should be carried out for at least 10 weeks or more (26).

This population is also vulnerable to a plethora of unfavorable health outcomes, including the risk of falls (6). So, we adapted the design and progression of exercise prescription of the Otago Exercise Program (OEP) (38) in respect to progressive strengthening and balance exercises. OEP is an evidence-based falls prevention program targeting community dwelling older adults which has yielded positive outcomes in overall improvement in physical function and a 35% decrease in falls among older persons with frailty (39). Further modifications of balance exercises were made to cater for functional training. These included improving performance in task accomplishment which often require multi-tasking when carrying out activities of daily living. The aim is to improve specific balance related ability such as regaining postural stability following perturbation, reaching upwards or downwards multiple times to collect household objects or even the ability to avoid obstacles while walking and talking (40). Lastly, the exercises were adjusted in accordance to the ACSM consensus recommendations for physical activity among older adults (41).

As for cognitive intervention, it is commonly delivered via technology such as computer-based training software or more recently commercialized packages for the use on smart devices (42). However, a non-technological and more traditional method of interactive cognitive training has also been shown to improve attention and memory among older Singapore population with frailty (23). We believed that this approach of cognitive training may be more sustainable and feasible as a low cost, community-based intervention for the purpose of our present study. Moreover, the targeted older persons for the present intervention expressed that they were not in favor of using technological gadgets in our needs assessment discussion.

The domains of cognition namely short-term memory, attention, information processing skills, perceptual organizational tasks, reasoning and logic, and problem-solving abilities were selected for cognitive training in our present intervention. This selection was based on the existing literature among older adults with cognitive impairment (23, 31, 43). We included “Pen to Paper” tasks such as “spot the difference,” mazes, matrix reasoning, and jigsaw puzzles. These tasks have been included as cognitive training resulting in enhanced cognitive function as it tackles multiple domains of cognition (visual perception, orientation perceptual reasoning, cognitive speed), and promotes an increase of brain reserve whilst preventing emotional distress which negatively impacts cognitive aging (23, 44). We deduce that this hands-on approach of cognitive training is not only effective in improving cognitive function but also beneficial in promoting social engagement as an activity of leisure.

For nutritional intervention, the existing interventions to address cognitive impairment or frailty consist of a wide range including a variety of supplementation, specialized diet and single or multi-nutrient intervention (33, 45, 46). As an alternative, dietary counseling, an inexpensive, and straightforward intervention and has been found to substantially reduce the risk of malnutrition among older adults (33). Hence, dietary counseling with education on healthy eating habits was opted as the nutritional intervention in our present study to allow older persons to sustain their healthy eating patterns as it is personalized to locally available and affordable produce.

Group directed intervention was the choice for the delivery of our present multi-domain intervention. This is because it is known to promote social participation, besides improving adherence to physical activity, psychological factors and social relationships; which further advocates that group-based intervention is key as a means of incorporating psychosocial well-being among older adults (47).

### Description of the WE-RISE Intervention (TiDieR Checklist)

#### Brief Name

The “WE-RISE” intervention stands for: Warga Emas - Resilient mInd and muScle Exercise. “Warga Emas” translates to Senior Citizens in the Malay language. The terms “resilient mind and muscle” was selected as we aim to reverse impaired physical and cognitive status of the older persons with respect to cognitive frailty.

#### Where: Intervention Location

Targeting community dwelling older adult population, we screened and recruited registered members of the Activity Centers for Older Persons; known locally as “Pusat Aktiviti Warga Emas” (PAWE) for the present study. PAWEs has been set up across the nation under the Malaysian Department of Social Welfare (48). These activity centers provide a social space promoting active participation and involvement of older persons within the community (48).

#### Intervention Providers

The exercise, cognitive and psychosocial component of the intervention was administered by a qualified physiotherapist with geriatric rehabilitation background (primary research coordinator). The physiotherapist in charge underwent training which included background to the adversity of cognitive frailty, rationale of the intervention, practical session of the intervention and participant safety moderated by Physiotherapist and Clinical Psychologist lecturers (research team members). The dietary component of the intervention was administered by a trained clinical dietician under the supervision of a Professor in Nutrition and Dietetics (research team member).

#### Procedure and Materials

In this randomized controlled study, participants were screened at baseline via face to face interview using a structured questionnaire for sociodemographic and clinical characteristics, psychosocial and functional status, cognitive function, quality of life and dietary intake. Anthropometry and physical performance measurements were objectively assessed. All assessments were conducted by qualified research assistants who were trained together. Inclusion criteria of this study were: Malaysian, community dwelling, aged 60 years and above, able to ambulate independently and classified to have cognitive pre-frailty or cognitive frailty. Older persons who were physically robust, diagnosed with terminal illnesses, major psychiatric illnesses, classified to have mild to moderate dementia, unable/refused to participate in the intervention, or already participating in other programs or on-going trial were excluded from the present study. The primary and secondary outcomes are as outlined.

##### Primary outcome: cognitive frailty

This study operationalized cognitive frailty as proposed by Kelaiditi et al. (17) using Fried's criteria as outlined in the Cardiovascular Health Study to define physical frailty and the Clinical Dementia Rating Scale (CDR) to define objective cognitive impairment. The presence of one or two of the Fried's criteria was defined as pre-frailty, whilst the presence of three or more was defined as frail and a score of 0.5 on the CDR is defined as mild cognitive impairment (MCI) (17). Collectively, participants who had a combination of pre-frailty/frailty and MCI were categorized as cognitive frailty (Table 1).

**Table 1:** Classification of cognitive frailty groups.

	**Criteria**	**Categorization**		
		**Robust**	**Cognitive pre-frailty**	**Cognitive frailty**
**Frailty** Fried et al. (6)	*Shrinking*: Unintentional weight loss of more than 5 kgs	0	1–2 Criteria	≥3 Criteria
	*Weakness*: Assessed with hand grip strength and adjusted for gender and body mass index against original cut-off points.			
	*Slowness*: Assessed with 5-meter gait speed test, adjusted for gender and height against original cut off points.			
	*Exhaustion*: Identified with two items from the CES-D scale.			
	*Low physical activity*: Identified by low scores of the PASE.			
**Cognitive impairment** Kelaiditi et al. (17)	Clinical Dementia Rating Scale score	0	0.5	0.5

*CES-D, Center for Epidemiologic Studies Depression Scale; PASE, Physical Activity Scale for Elderly*.

##### Secondary outcomes

Sociodemographic information, Clinical Characteristics and Lifestyle: Sociodemographic and lifestyle variables included age, gender, level of education, ethnicity, marital status, status of employment, income status, alcohol consumption and smoking history. Clinical characteristics included history of falls, family history of dementia and history of chronic diseases.

Physical Fitness and Functional Status: The senior fitness test by (49) was used to measure physical fitness. Thirty-Second Chair Stand test for lower body strength assessment; Back Scratch test for upper body flexibility assessment; Sit-and-Reach test for lower body flexibility; Timed Up and Go test for mobility and balance; 6-meter Gait Speed test for gait speed; and the 2-Min Step test to assess cardiovascular fitness and endurance. Functional status was assessed by level of independence based on the Instrumental Activities of Daily Living (IADL) (50).

Cognitive Function: The Mini Mental Examination State (MMSE) was used to assess global cognition (51). The Rey Auditory Verbal Learning Test (RAVLT) was used to detect short-term verbal memory, working memory, verbal learning and declarative memory (52). The Digit Span test, originally a test from Weschler Adult Intelligence Scale (WAIS) assessed memory, attention and concentration (53). The Trails Making Test (TMT) was used to assess processing speed and mental flexibility (54).

Nutritional Status: Anthropometric measurements and body composition were used as indicators of nutritional status. Length of arm demi-span, mid-upper arm circumference, waist circumference, hip circumference, and calf circumference were measured using the Lufkin^®^ W606PM Anthropometric Tape Measure. Body composition including body mass index, metabolic age, fat percentage (%), fat mass (kg), fat free mass (kg), and muscle mass (kg) was measured using the Tanita^®^ TBF-400 Total Body Composition Analyzer.

Dietary Intake: Dietary intake was assessed using the Dietary Habits Questionnaire (DHQ) to estimate the overall dietary intake, usual dietary habits and the mean intake of various types of nutrients (55). The food intake will be analyzed using Nutritionist-Pro software to estimate the total calories, macronutrients and micronutrients intake.

Psychosocial Status: The Geriatric Depression Scale-15 (GDS) was used to detect symptoms of depression (56). Domains of functioning and disability was assessed using the WHODAS 2.0 (57).

Quality of Life: The 15-D was used as a measure of health-related quality of life (HRQoL). It describes the participants' self-perception of the following domains: mobility, vision, hearing, breathing, sleeping, eating, speech, excretion, usual activities, mental function, discomfort and symptoms, depression, distress, vitality, and sexual health (58).

##### Randomization

**Figure 1:**
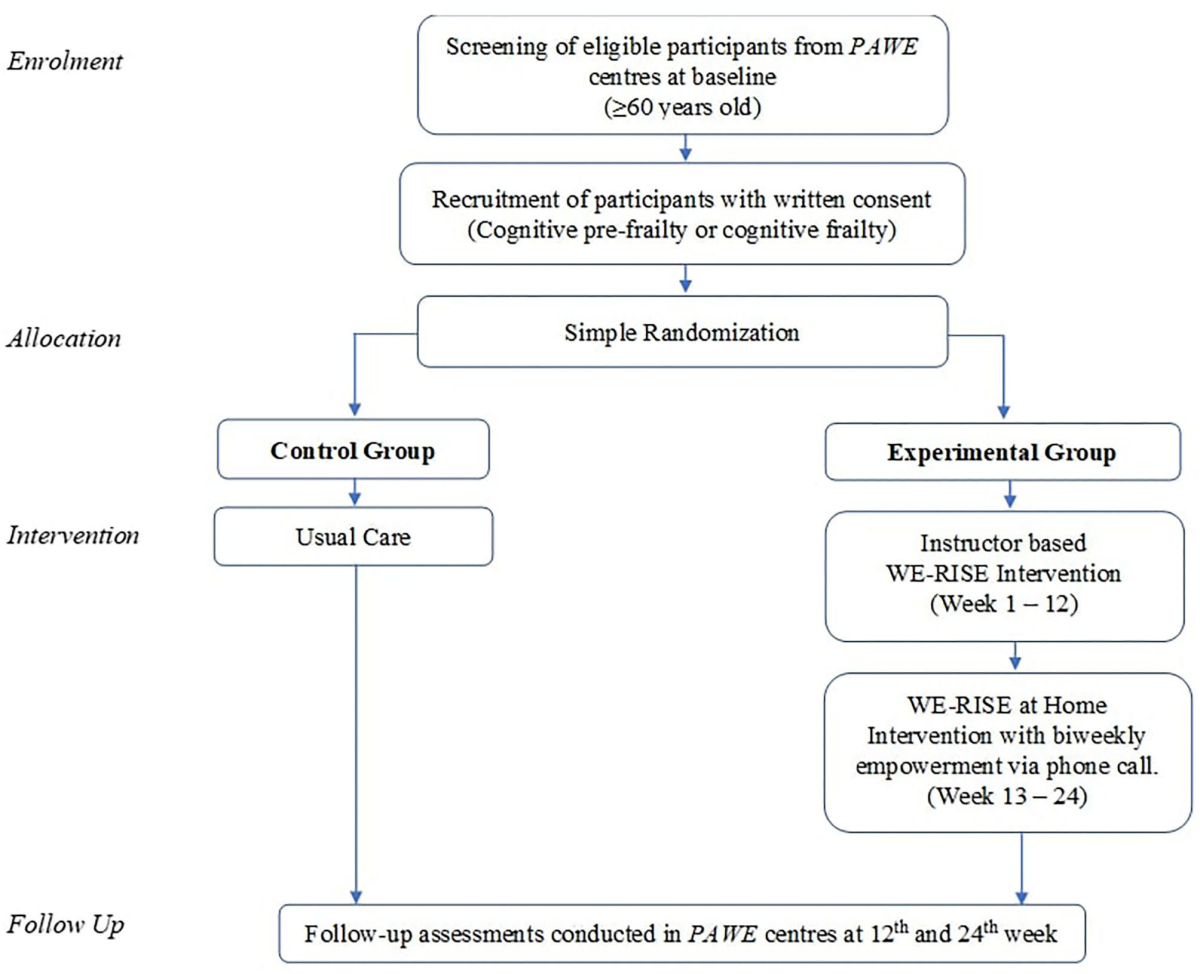
Flow diagram of the WE-RISE trial.

Participants with cognitive pre-frailty or cognitive frailty were randomized into intervention and control groups. Simple randomization was executed using the Research Randomizer computer program by the primary research coordinator (59). The trial flow is as illustrated in Figure 1. In this single blinded study, research assistants involved in data collection for the baseline and follow up outcome measures to prevent bias were blinded of the groups. Primary research coordinator was not involved in data collection. In order to minimize contamination between the intervention and control groups, an arrangement was made with the participating PAWEs to allow private and scheduled use of the facility on days that are not open for member activities.

##### Intervention

*Experimental Group* The 24-week (6 months) intervention was divided into two phases. Phase 1 comprised of the first 12 weeks whereby an instructor guided group-based intervention was conducted at PAWE centers twice a week for a duration of 90 min per session. Phase 2 comprised of the following 12 weeks whereby the “WE-RISE at Home” is independently carried out by the participants as a home-based intervention.

At the commencement of the WE-RISE intervention, the therapist explained each component of the intervention and what was expected during each intervention session. The 12 weeks of intervention was divided into 3 parts: Level 1 – week 1 – 4, Level 2 - week 5 to 8 and Level 3 – week 9 to 12 with increasing level of intensity for the exercises and cognitive training.

*Multi-component exercise training*. The frequency, intensity, time and type (FITT) principle for this study was established based on best available recommendation for effective exercise interventions among community dwelling older persons with frailty (26). The FITT framework also corroborates with the gold standard guidelines established by the American College of Sports Medicine (ACSM) for exercise prescription in older persons (41). The exercise regime of this intervention is as outlined in detail in Tables 2–5. The exercise training begins and ends with flexibility training consisting of mobility and dynamic stretching of major joints as a warm-up and cool down. This component of the exercise program remains unchanged throughout Level 1–3 (Table 2). Aerobic training is administered through dance aerobic sessions. The dance aerobics will largely comprise of active movements such as stationary march, front and backward march, side walking with a turn, toe steps, in combination with upper and lower limb movements (Table 3). Progressive resistance training targeted upper limb, lower limb and body weight exercises to improve overall muscle strength. The exercises carried out from level 1 to level 3 are maintained throughout with variation in level of resistance and repetitions per set for each exercise. Resistance in this intervention is provided with the usage of weight cuffs (Table 4). The balance and coordination training include multi-task exercises which recruit not just postural control and strength muscles, but also requires cognitive processing (Table 5). A PVC elastic ball is used for part of the balance exercises.

**Table 2:** Flexibility training program.

	**Level 1**	**Level 2**	**Level 3**
*Flexibility: joint mobility & dynamic stretching*	Duration of exercise:10 min		
Head and Neck Shoulder and Arm, Trunk	Repetition: 8 counts for each plane of motion for mobility		
Hip and Knee, Ankle	Stretching: Each stretch sustained for 8 s in each plane of motion		

**Table 3:** Aerobic training program.

	**Level 1**	**Level 2**	**Level 3**
*Aerobic training*	Duration of Exercise: 15 min		
	Type: Dance Aerobics		
	Intensity: RPE 3–5; moderate intensity		Intensity: RPE 6–8; vigorous intensity.

*RPE, Rate of Perceived Exertion*.

**Table 4:** Progressive resistance training program.

	**Level 1**	**Level 2**	**Level 3**
*Progressive resistance training*	Duration of exercise: 30–40 min		
	Number of muscle groups: 8–10		
Upper body strength	Repetition: 8 Number of sets: 3 Weight cuff: 0.5 kg around wrist.	Repetition: 10Number of sets: 3 Weight cuff: 1.0 kg around wrist.	Repetition: 12 Number of sets: 3 Weight cuff: 2.0 kg around wrist.
Shoulder press			
Lateral shoulder raise			
Front shoulder raise			
Triceps extension			
Bicep curl			
Lower body strength	Repetition: 8 Number of sets: 3 Weight cuff: 0.5 kg around ankle.	Repetition: 10Number of sets: 3 Weight cuff: 1.0 kg around ankle.	Repetition: 12 Number of sets: 3 Weight cuff: 2.0 kg around ankle.
Seated knee raise			
Seated knee extension			
Standing hamstring curl			
Standing lateral leg lift			
Calf raises			
Heel raises			
Sit to stand Half-squat (with support)	Repetition: 8 Number of sets: 3 Weight cuff: 0.5 kg around wrist.	Repetition: 10 Number of sets: 3 Weight cuff: 1.0 kg around wrist.	Repetition: 12 Number of sets: 3 Weight cuff: 2.0 kg around wrist.

**Table 5:** Balance and coordination training program.

	**Level 1**	**Level 2**	**Level 3**
Weight shifting	Side to side, frontward to backwards, 8 reps, with support.	Side to side, frontward to backwards, 10 reps, no support.	Side to side, frontward to backwards, 12 reps, no support.
Single leg stand	10 s each leg, with support.	10 s each leg, without support.	10 s each leg, without support.
Semi-tandem stand	10 s, eyes open, with support	10 s, eyes open/closed, with support.	10 s, eyes open/closed, without support.
Tandem stand	10 s, eyes open, with support	10 s, eyes open/closed, with support.	10 s, eyes open/closed, without support.
Sideways walking	10 steps, 4 reps, with support.	10 steps, 4 reps, without support.	10 steps, 4 reps, without support.
Backward walking	10 steps, 4 reps, with support.	10 steps, 4 reps, without support.	10 steps, 4 reps, without support.
Walking with a turn	Walk and turn in the figure 8	Walk and turn in the figure 8	Walk and turn in the figure 8
Heel walking	10 steps, 4 reps, with support.	10 steps, 4 reps, without support.	10 steps, 4 reps, without support.
Toe walking	10 steps, 4 reps, with support.	10 steps, 4 reps, without support.	10 steps, 4 reps, without support.
Ball activity	Ball dribbling and throwing upwards & single direction ball throwing with partner.	Ball dribbling and throwing upwards with one hand; ball throwing/catching in different directions in a stationary circle.	Ball dribbling and throwing upwards with one hand; throwing/catching ball in different directions while rotating in circle.
Tandem walk	10 steps, 4 reps, with support.	10 steps, 4 reps, with support.	10 steps, 4 reps, without support.
Slalom walking	Slalom walk around stationary obstacles.	Slalom walk around stationary obstacles in haphazard directions.	Slalom walk around while picking up stationary obstacles in haphazard directions.
Coordination training	Hand eye coordination training		

There may be a risk of injury involved while participating in physical activity. Older adults are susceptible to injuries such as sprains, repetitive strain, falls, muscle fatigue, muscle cramps or may be hesitant due to fear of injury (60). To overcome these adverse outcomes, safety is ensured at all times by carrying out the activity in the presence of support (chair or wall). Intermittent breaks are provided throughout the exercise regime and hydration is a priority. Participants are arranged in a manner whereby each individual is in the view of the instructor and vice versa. Participants will be briefed that they may experience some form of delayed onset muscle soreness (DOMS) due to muscle adaptation to exercise which is normal and will cease (61). Participants are also advised to rest should they feel pain, discomfort or intolerance to the exercises.

*Cognitive training*. The activities in the intervention will include “paper and pencil tasks,” puzzle activities, memory games, “spot the difference,” coloring activities, matrix reasoning, maze activities and sorting activities as outlined in Table 6. Materials for the cognitive training include, stationary, cognitive challenge worksheets, jigsaw puzzle, memory cards, colored ice cream sticks and colored toothpicks. The level of difficulty will be increased each month to further challenge the participants' cognitive function.

**Table 6:** Outline and description of cognitive training program.

**Activity**	**Domain of Cognition**	**Description of Activity**	**Levels of Difficulty**
“Getting ‘jiggy’ with it” - Jigsaw puzzle	Visuospatial reasoning & working memory	Assembling jigsaw puzzle.	Level 1: 6 Piece puzzle Level 2: 6 Piece & 9 Piece puzzle. Level 3: 6,9- and 12-piece puzzle.
“Let's get it sorted” - Sorting game	Executive function & cognitive flexibility	Sorting different objects by color first and then progressing to sorting by name of color.	Level 1: Ice cream stick and sort only 2 colors. Level 2: Ice cream Stick sort but color of stick or tub. Level 3: Matchstick sort by color of stick and color of tub.
“It's not the same!” - Spot the difference	Attention & processing speed	Two similar pictures are printed on a single sheet. Participants are to spot and circle 10 differences between the two.	Increasing level of complexity of the worksheets provided.
“Get out!” - Maze Activity	Problem solving skills & reaction time	Maze activities printed on paper. Participants are to find their way out of the maze with a pencil.	Increasing level of complexity of the mazes provided.
“What did you see?” - Recall activity	Short term memory	Show a picture for 30 s, remember all the objects within the picture and list as many as possible.	Level 1: 5 objects Level 2: 10 objects Level 3: 15 objects
“Jog that memory” - Memory games with cards.	Reaction time, attention & processing speed	This is a card-based memory game. A deck of cards containing paired pictures is used to play memory-based games such as “snap” or quick pairing.	Level 1: 2 players, 1 deck of cards Level 2: 4 players, 2 deck of cards Level 3: All participants together, combination of 3 different decks.
“Color codes”	Concentration & matrix reasoning	These games are a combination of colors and shapes on paper. Simple sudoku type activity will be given, instead of numbers with shapes of different colors.	Level 1: Only 1 empty spot in each row. Level 2: 2 empty spots in each row. Level 3: Multiple missing spots to be filled.

*Dietary Counseling*. A qualified clinical dietitian conducts a one-off group dietary counseling session which includes the distribution of a dietary information pamphlet to promote physical and cognitive well-being. The pamphlet includes meal by meal guidance and healthy eating habits to be practiced pre- and post- exercise. Participants are encouraged to contact the dietician via phone call should they have any queries.

*Psychosocial support*. Intervention is conducted as a group-based activity which are enjoyable and interesting with constant facilitation. Elements of group-based sessions encompass friendly interaction, support based communication and the establishment of a non-threatening environment to ensure adherence to intervention and improve self-esteem (62). It should be noted that, all activities in the multi-domain intervention excludes competitive components.

*Home based program – “WE-RISE at home”*. Following the completion of the 12-week center based WE-RISE program, the participants were instructed to carry out the intervention in the comfort of their home independently twice a week for 12 weeks. A “WE-RISE at Home” packet containing an activity manual, dietary guideline, 12 sheets of cognitive training activities, 2 sets of jigsaw puzzle, a ball, a pair of 2 kg weight cuffs and stationery, is given to each participant. The manual contains illustrated and written, step-by-step instructions on how to carry out the exercises while ensuring safety at all times. Each participant was provided with a calendar in the manual with scheduled dates to carry out the home program and they were asked to tick the date boxes after completing each session which served as a log for record keeping. Although participants were to stick to the exercises and cognitive activity included in the “WE-RISE at Home”; they were given the autonomy to decide the type of aerobic exercise they preferred to carry out (a choice of: dance aerobics, brisk walking, stationary or mobile jogging, stationary march). They were also able to select which cognitive activity they felt like tackling for each session. Empowerment strategies have been found to play a vital role in bringing forth positive health outcomes and making informed health decisions (63). Participants were contacted every 2 weeks via phone call as an empowerment method aimed to provide social support, motivation, promote positive health behaviors and create awareness of self-efficacy. In addition, it also enabled monitoring of the participants' compliance to the intervention and their health status as the intervention progressed.

*Control group*. The control group in this study received usual care and participate in PAWE weekly conducted community activities such as cooking classes, karaoke, arts and craft, chair exercises, with no changes made to their habitual routine of daily life.

#### Tailoring

This was a standardized group targeted program, with no individual tailoring.

#### Adherence/Fidelity

To ensure the intervention is conveyed as per the protocol, the physiotherapist in charge was observed and assessed by researchers of physiotherapy and clinical psychology background. The factors taken into assessment were appropriate exercise prescription for older persons with cognitively frailty, safety of the intervention delivery, manner of intervention delivery and adherence to the specified protocol. Fidelity of participants to the intervention was monitored by attendance logs of each participant for each intervention session. For the WE-RISE at Home, the number of completed session is self-reported by the participants over the biweekly phone calls with therapist as well as marked in the calendar within the activity manual. Additionally, elements of motivation, psychological and practical support, goal setting and focus on independence are incorporated as adherence measures older adults with frailty and cognitive impairment (64, 65).

#### Data Analysis

All statistical analyses will be carried out using the Statistical Package for Social Sciences (SPSS) software, version 23.0. An alpha level of (0.05) was considered for all the statistical tests used in the study. Two-sided *p* values of (0.05) and (80%) power will be statistically significant. Results of the randomized controlled trial will be analyzed using repeated measures analysis of variance for pre-test (Baseline Scores) and post-test (3rd month, 6^th^ month) for experimental and active control group. *Post-hoc* analysis will be conducted using Benferonni correction. Analysis will include nutritional assessment, cognitive frailty outcome measures, cognitive assessments, physical function assessments and quality of life.

## Discussion

To the best of our knowledge, this multi-domain intervention incorporating cognitive, physical, nutritional and psychosocial domains specifically targeting the potential reversibility of cognitive frailty will be the first of its kind in Malaysia, as compared to existing interventions which address physical frailty and cognitive impairment separately among community dwelling older adults. The description of the WE-RISE intervention was reported using the TIDieR (66) to enable smooth replication into practice should it be found to be effective in addressing cognitive frailty.

We employed strategies that were found to be effective in the management of physical frailty and cognitive impairment in the hopes that the same outcomes will be obtained when the conditions co-exist simultaneously. There is an apparent gap in evidence regarding the existence of interventions in the ‘real-world’ setting (67). The novelty of this intervention is that the multiple domains of the intervention are tackled in a single session and it is executed in a “real-world” setting. This form of delivery overcomes the challenges faced when disseminating evidence-based interventions that are usually conducted in controlled and optimum environments to practice (67). The WE-RISE intervention is designed to adapt to the local setting, hence the translation to real-world practice is anticipated to be less challenging and sustainable. Likewise, the intervention is progressive in nature and is simple to comprehend and adhere to. The WE-RISE at Home program strongly promotes the continuation of self-management following the instructed sessions at the comforts of their own home. The elements of social engagement via phone call with instructor, autonomy to select the activities of choice at home and setting targets to achieve for each session are promising components that heighten motivation to carry out activities independently (65).

The WE-RISE intervention hopes to provide easier accessibility whilst enabling “age-friendly” health care delivery. If the intervention is found to be effective, the community-based nature of the intervention delivery may also be of lower cost as compared to the process of obtaining standard healthcare. Furthermore, it is not specific to the subset of older persons from lower socioeconomics but can also be applied amongst all walks of life and be practiced as a preventative strategy. The effectiveness and cost effectiveness of the WE-RISE intervention as compared to usual care on the reversal of cognitive frailty status among community dwelling older adults will be reported at the end of the trial. It is hoped that WE-RISE which is a multi-component and domain intervention will not only be beneficial in addressing cognitive frailty but also physical activity and general health in older persons.

In conclusion, we hope the address the need for a comprehensive and feasible intervention which is also sustainable in terms of delivery and cost for the well-being of older persons with cognitive frailty. The WE-RISE is versatile and can be administered within the community as well as a hospital or nursing home setting by most exercise instructors or primary healthcare practitioners with training.

## Trial Registration

This trial was registered in the Australian New Zealand Clinical Trials Registry (ANZCTR) on the 29th of July 2019. Registration number: ACTRN12619001055190.

## Data Availability Statement

The original contributions presented in the study are included in the article/supplementary materials, further inquiries can be directed to the corresponding author/s.

## Ethics Statement

The studies involving human participants were reviewed and approved by Research Ethics Committee of Universiti Kebangsaan Malaysia (UKM PPI/111/8/JEP-2018-558) Department of Social Welfare Malaysia (MyResearch Reference: JKMM 100/12/5/2: 2018/405). The patients/participants provided their written informed consent to participate in this study.

## Author Contributions

RM: conceptualization, methodology, project administration, resources, and writing – original draft. DS: conceptualization, methodology, project administration, resources, supervision, writing – review & editing, and funding acquisition. SS: project administration, resources, supervision, writing – review & editing, and funding acquisition. PS: methodology, supervision, and writing – review & editing. All authors contributed to the article and approved the submitted version.

## Conflict of Interest

The authors declare that the research was conducted in the absence of any commercial or financial relationships that could be construed as a potential conflict of interest.
